# Cumulative Strain and Improvement Mechanisms of Soil Reinforced by Xanthan Gum Biopolymer Under Traffic Loading

**DOI:** 10.3390/polym16243500

**Published:** 2024-12-16

**Authors:** Liu Yang, Lingshi An, Kuangyu Yan, Gaofeng Du

**Affiliations:** 1 Hunan Mine Carbon Sequestration and Sink Enhancement Engineering Technology Research Center, Changsha 410151, China; 2 Key Laboratory of Metallogenic Prediction of Nonferrous Metals and Geological Environment Monitoring, Central South University, Ministry of Education, Changsha 410083, China; 3 Department of Natural Resources, Hunan Vocational College of Engineering, Changsha 410151, China; 4 School of Civil and Architecture Engineering, East China University of Technology, Nanchang 330013, China

**Keywords:** xanthan gum biopolymer, cumulative strain, improvement mechanisms, SEM, traffic loading

## Abstract

As is widely accepted, cumulative strain and improvement mechanisms of stabilized soil are critical factors for the long-term reliable operation of expressways and high-speed railways. Based on relevant research findings, xanthan gum biopolymer is regarded as a green and environmentally friendly curing agent in comparison to traditional stabilizers, such as cement, lime, and fly ash. However, little attention has been devoted to the cumulative strain and improvement mechanisms of soil reinforced by xanthan gum biopolymer under traffic loading. In the current study, a series of laboratory tests, including cyclic triaxial tests and scanning electron microscopy (SEM) tests, were performed to investigate this issue in more detail. The influences of xanthan gum biopolymer content, curing time, moisture content, confining pressure, and cyclic stress amplitude on cumulative strain were analyzed. In addition, the cumulative strain model was proposed to provide a good description of experimental data. Finally, the microscopic structure of soil reinforced by xanthan gum biopolymer was analyzed to discuss the improvement mechanisms. The results show that the cumulative strain is strongly influenced by xanthan gum biopolymer content. For a given number of loading cycles, the greater the confining pressure, the smaller the cumulative strain. The calculated results of the cumulative strain model show a good agreement with test data. The “flocculent” hydrogel can form a denser structure and greater bonding strength in comparison to the “branch-like” and “net-like” hydrogels.

## 1. Introduction

It is common knowledge that soil that exhibits several adverse engineering properties, such as loose structure [1,2], low strength [3,4], and high compressibility [5,6], poses significant challenges in engineering applications, as these unsatisfactory engineering characteristics can lead to considerable deformation under traffic loading, compromising the stability and integrity of the subgrade. Consequently, the improvement of soil has attracted significant attention among researchers across various disciplines, including civil engineering and environmental science [7]. The improvement techniques can be broadly categorized into physical and chemical approaches [8,9]. At present, cement stabilization is the most extensively used technique [10,11]. Nevertheless, the cement improvement technique is associated with a series of issues, including high carbon dioxide emissions [12,13], substantial natural resource consumption [14,15], and severe environmental pollution [16,17]. For this reason, it is imperative to explore a green, low-carbon, and efficient environmentally friendly curing agent for soil stabilization.

It is worth noting that xanthan gum biopolymer is a polysaccharide produced by Xanthomonas campestris through the fermentation of glucose or sucrose. It exhibits excellent adhesive and pseudoplastic properties, even at low concentrations [18]. Additionally, its rigid, rod-shaped helical structure provides it with remarkable stability, which makes it less susceptible to degradation caused by environmental factors such as temperature and acidity. Upon interacting with water, xanthan gum biopolymer forms hydrogen bonds that cement the soil particles together, reducing pore space and enhancing adhesion, thus effectively increasing soil stiffness and bearing capacity. As a consequence, xanthan gum biopolymer is regarded as a green and environmentally friendly curing agent, widely used in soil improvement over the past few years [19,20]. Azimi [21] pointed out that the optimum content of xanthan gum biopolymer for soil stabilization was 1.5%. Furthermore, extending the curing time from 7 days to 28 days resulted in a significant improvement in the unconfined compressive strength (UCS) of the treated soil. Tabarsa [22] discovered that xanthan gum biopolymer greatly enhanced soil’s mechanical properties, with UCS improvements ranging from 177% to 259%, depending on dry unit weight. The cohesion increased by 463% to 563%, and the internal friction angle rose by 41% to 51%. These improvements occurred within a 7-day curing period, demonstrating xanthan gum biopolymer’s potential for rapid strength applications in emergency construction. Bagheri [23] observed that the soil’s compressive strength increased by 2.5 times with 2% xanthan gum biopolymer. CU triaxial tests showed that water absorption reduced strength and shearing parameters. UU triaxial tests on dried samples indicated substantial improvements in strength and cohesion from xanthan gum biopolymer. Despite UCS values decreasing with wetting/drying cycles, xanthan gum biopolymer-treated samples exhibited higher strength than untreated soil. Ramesh [24] found that even at just 0.5% by mass, xanthan gum biopolymer can greatly reduce accumulated axial strain. For instance, using 0.5% xanthan gum biopolymer reduced cyclic axial strains to 2.9% under challenging conditions, which caused untreated soil to fail immediately. According to the research by Li [25], the treatment of soil with xanthan gum biopolymer resulted in the highest strength when compared to treatments with Ca-alginate and agar gum. This superior strength was primarily due to the unique properties of xanthan gum biopolymer, which can create gel matrices within the soil.

In recent years, with the rapid development of the economy, a large number of major transportation projects such as highways and high-speed railways have been constructed all over the world. Numerous investigations have demonstrated that the cumulative strain of stabilized soil under traffic loading is a critical factor for the long-term reliable operation of expressways and high-speed railways [26,27]. Hejazi [28] discovered that while the amplitudes of principal stress rotation stayed consistent during loading, the amplitudes of principal strain rotation decreased to negligible levels with increasing cycles. This implied that shear strains became insignificant at high cycle counts, with deformation primarily axial. The study showed a systematic increase in axial deformation relative to shear strain, at a slower pace for specimens with initial static shear stress. S. R. [29] observed that permanent strain was decreased by fiber insertion, and increased confining pressure also led to a reduction. Soil with 1% fiber content showed about a 50% decrease in permanent strain for all confining pressures. Polypropylene fibers enhanced the soil’s resistance to deformation. More fibers increase interactions, forming a denser network that further reduces permanent strain. An [30] pointed out that the cumulative strain decreased almost linearly with increasing curing time. This indicated that longer curing time resulted in a denser and more cohesive soil skeleton, which can better resist deformation under traffic loading. Santos [31] discovered that the observed mechanical behavior was largely due to a reduction in soil matric suction from increased moisture content. With higher initial moisture content, particle arrangement shifted and the air phase was occluded, decreasing soil suction and increasing plastic deformations. Additionally, post-compaction moisture addition diminished capillarity effects and soil suction, leading to more total permanent deformations. According to the research by Chandra [32], the net deformation was found by subtracting the natural subgrade layer’s permanent deformation from the total permanent deformation. A significant difference in permanent deformation was observed between treated and untreated soil in the stabilized subgrade layer. Enzyme treatment resulted in a 33% reduction in permanent deformation after 30 loading cycles.

It should be noted that the SEM technique has been extensively used to investigate the microstructure of stabilized soil to clarify the improvement mechanisms [33,34]. Muhammad [35] pointed out that when xanthan gum biopolymer was at 1.5% or higher, molecules filled voids and coated soil grains with dense hydrogels, leading to dense clay flocculation and improved strength. The SEM micrograph’s appearance changed significantly due to the biopolymer gel cover. Exceeding 1.5% resulted in a more conglomerated soil matrix with larger voids. Rauf [36] discovered that microscopic analysis revealed a correlation between fiber content and improved soil strength. In the case of 3% cement, 0.25% polypropylene fiber did not significantly strengthen the soil. However, 1% fiber addition stabilized the soil more effectively. The fibers bound the soil–cement matrix, forming a robust network that improved resistance to deformation and fracture, resulting in a noticeable improvement effect. Vishweshwara [37] observed that the gel threads of acacia gum biopolymer improved the soil matrix, leading to enhanced strength. The soil matrix became stronger as curing progressed, with fibrous linkages stiffening during drying. Moreover, acacia gum biopolymer adsorbed onto clay soil surfaces, creating a solid, cohesive mass over time. Arabani [38] discovered that the sticky gel formed by nano clay improved adhesion between soil and fibers, while also acting as a filler by absorbing water between soil particles. The adhesion increase was due to the viscous gel adhering more strongly than double-layer water. Barley fibers, more porous than synthetic ones, allowed nanoparticles to infiltrate and improved soil contact and adhesion. According to the research by Shen [39], in the polyurethane-treated soil, the presence of floating adhesive, surface attachments, and adhesive between particles was evident. At lower polyurethane contents, the polyurethane primarily manifested as floating adhesive and surface attachments on the soil particles. As the polyurethane content increased, its role evolved from merely providing surface attachments to acting as a strong adhesive between particles. 

However, there is a lack of understanding regarding the cumulative strain and improvement mechanisms of soil reinforced by xanthan gum biopolymer under traffic loading. Given this, a series of cyclic triaxial tests and SEM tests were carried out to investigate this issue in more detail. In this paper, the impacts of xanthan gum biopolymer content, curing time, moisture content, confining pressure, and cyclic stress amplitude on the cumulative strain were evaluated. In addition, the cumulative strain model was proposed to describe the experimental data. Finally, the microscopic structure of soil reinforced by xanthan gum biopolymer was studied to discuss the improvement mechanisms.

## 2. Laboratory Test Program

### 2.1. Materials and Experimental Instrument

**Figure 1:**
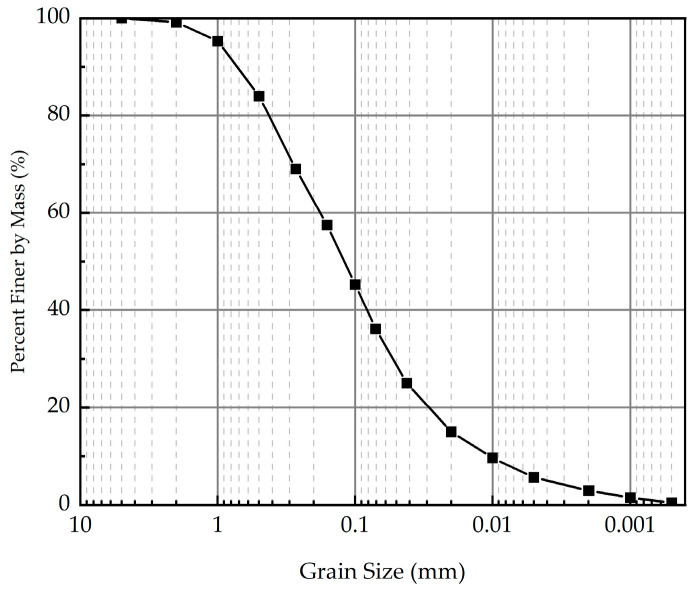
The grain-size distribution curve of soil.

The soil used in the current study was obtained from the embankment along the Qingdao-Lanzhou Expressway, China. The physical properties of soil, such as moisture content, maximum dry density, liquid limit, and plastic limit are listed in Table 1. The grain-size distribution curve of soil is presented in Figure 1. 

**Figure 2:**
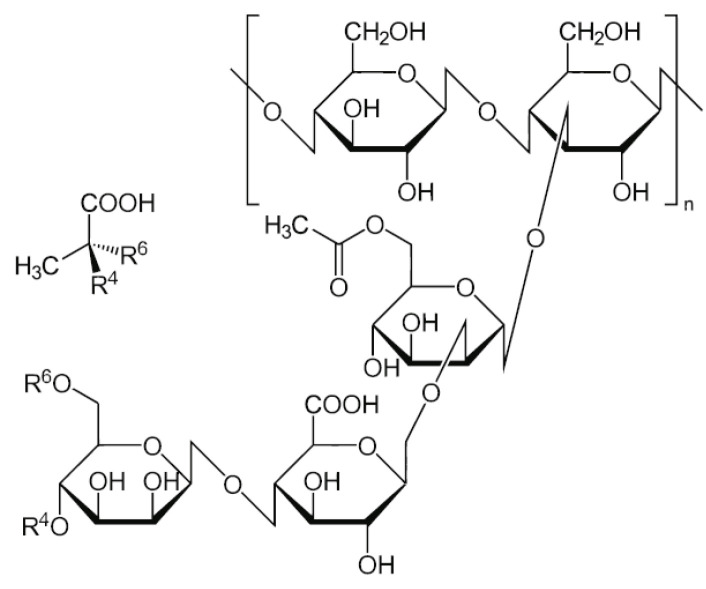
The molecular structure of xanthan gum biopolymer [40].

**Figure 3:**
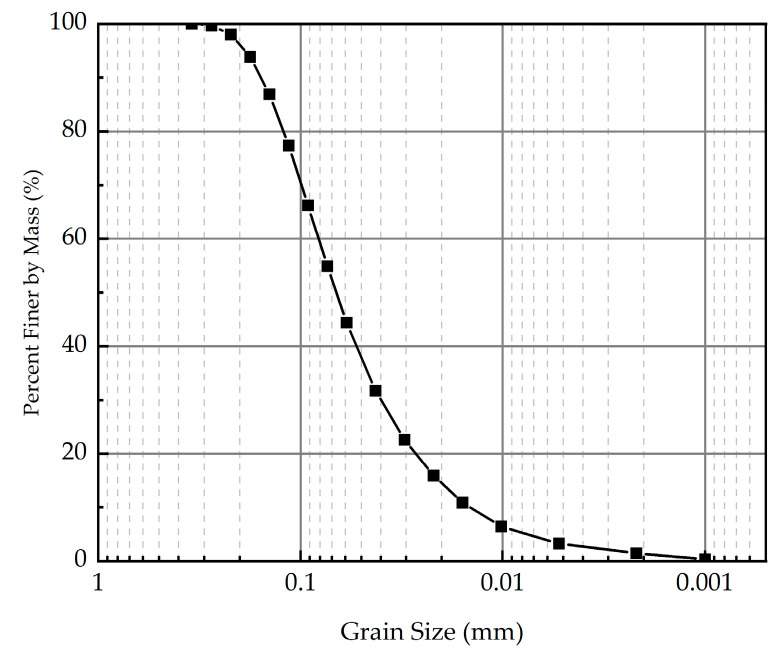
The grain-size distribution curve of xanthan gum biopolymer.

Xanthan gum biopolymer, characterized as a water-soluble anionic polysaccharide, is generally composed of D-glucose, D-mannose, D-glucuronic acid, acetyl groups, and pyruvate. The molecular structure of xanthan gum biopolymer is depicted in Figure 2. According to the previous studies [18,25], compared to traditional curing agents, such as cement, lime, and fly ash, xanthan gum biopolymer has the advantages of being environmentally friendly, highly efficient, and low-carbon. For this reason, xanthan gum biopolymer was adopted as an environmentally friendly curing agent in this research. The xanthan gum biopolymer used in this test was produced by Deosen Biochemical (Ordos) Ltd (Nei Mongol, China). The basic properties of xanthan gum biopolymer are presented in Table 2. The grain-size distribution curve of xanthan gum biopolymer is presented in Figure 3.

The experimental instrument of the cyclic triaxial test is the MTS triaxial testing system. The manufacturer is MTS Systems Corporation, Eden Prairie, MN, USA, and the equipment series is MTS 793. The MTS triaxial testing system is equipped with an axial loading system, a confining pressure loading system, an automatic measuring system, and a data acquisition system. Various waveforms, including sine, triangle, and user-defined waves, can be applied to the specimens. The maximum axial load, maximum axial displacement, maximum confining pressure, and maximum frequency are 250 kN, 80 mm, 20 MPa, and 20 Hz, respectively.

The laboratory apparatus for the SEM test is the field emission scanning electron microscope. The manufacturer is Carl Zeiss AG Co. Ltd., Oberkochen, Germany, and the equipment series is Gemini SEM 300. The field emission scanning electron microscope is equipped with a five-axis fully automatic motorized sample stage, an in-lens secondary electron detector within the objective lens, a variable pressure secondary electron detector in the sample chamber, and a cathodoluminescence detector. The resolution is 1.0 nm, the magnification ranges from 10 to 1,000,000, and the sample stage movement range is 125 mm (X direction), 125 mm (Y direction), 50 mm (Z direction), −10° to 90° (tilt), and 360° (rotation).

### 2.2. Specimen Preparation and Experimental Procedure

The procedures of preparing specimens were performed according to the Chinese Standard for Geotechnical Testing Method (GB/T 50123-2019) [41], and were designed as follows: (1) The soil was put in an oven at 105 °C for 24 h to ensure it was completely dried. (2) According to the xanthan gum biopolymer content and moisture content specified in the experimental scheme summarized in Table 3, dried soil, xanthan gum biopolymer, and distilled water were thoroughly mixed. (3) To facilitate convenient separation of the specimen, a film of aircraft hydraulic oil was coated on the internal surface of the sample-making mold, which was 50 mm in diameter and 100 mm in height. (4) The mixture was put into the sample-making mold in the same five layers and each layer was compacted. (5) The specimen was separated from the sample-making mold, and was wrapped with cling film immediately. (6) The specimen was placed in a curing box, where the humidity was set to the moisture content specified in the experimental scheme. (7) The specimens were made in batches to ensure their comparability.

**Figure 4:**
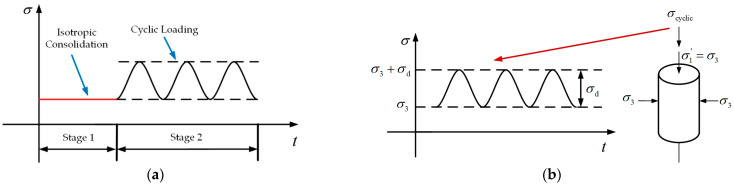
Test procedures: (**a**) Workflow of experiment; (**b**) Load-on pattern of one-way cyclic loading.

**Figure 5:**
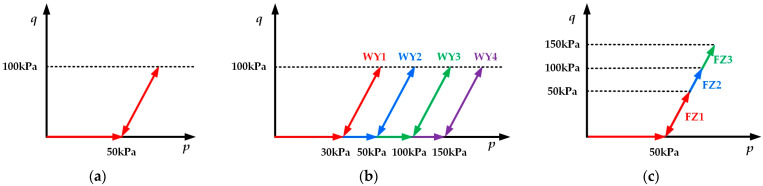
The applied stress paths of cyclic triaxial tests: (**a**) CL1-CL6, LQ1-LQ5, and HSL1-HSL4; (**b**) WY1-WY4; (**c**) FZ1-FZ3.

Based on relevant research findings [26,27], the cyclic triaxial tests were performed under consolidated undrained conditions. The experimental procedure can be divided into two stages: the isotropic consolidation stage and the cyclic loading stage, as presented in Figure 4. The applied stress paths of cyclic triaxial tests are indicated in Figure 5.

(1) In the isotropic consolidation stage, the specimens were subjected to isotropic consolidation under the confining pressures required by the experimental scheme summarized in Table 3. During this stage, *p* equaled the confining pressure, and *q* equaled zero, as depicted in Figure 5. According to the previous studies [29,42], the confining pressures of 50 kPa, 100 kPa, and 150 kPa can represent the actual confining pressures. Hence, in order to investigate the impact of confining pressure on cumulative strain in more detail, the confining pressures used in this paper were 30 kPa, 50 kPa, 100 kPa, and 150 kPa.

(2) In the cyclic loading stage, a series of long-term, low-level cyclic triaxial tests were conducted. The cyclic loading was a one-way loading applied to specimens only in the axial direction, and the lateral stress remained constant throughout the testing period. A complete one-way cyclic loading consisted of a loading phase and an unloading phase, with the total loading amplitude at the end of unloading phase equaled to the confining pressure. This meant that during the loading phase, the total loading would not be less than the confining pressure, ensuring the specimen remained in a compressed state. During this stage, *p* equaled the confining pressure, and *q* equaled the cyclic stress amplitude, as depicted in Figure 5. Based on relevant research findings [43,44], it was observed that the cyclic stress amplitude was typically between 40 kPa and 120 kPa. In order to evaluate the influence of cyclic stress amplitude on cumulative strain in more detail, the cyclic stress amplitudes used in this paper were 50 kPa, 100 kPa, and 150 kPa. In addition, the loading frequency used in this paper was 1 Hz. 

The process of SEM testing is as follows: (1) To capture the structural features, a soil sample of approximately 1 cm^3^ was obtained using a fine steel wire in the soil deposition direction, while avoiding smooth surfaces, with the sampling point at the center of the specimen. (2) To prepare for scanning, a rubber ball was used to clear the soil particles from the sample’s surface. It was then sprayed with gold to avoid potential charge effects before testing, followed by a quick transfer to the field emission scanning electron microscope for testing. Note that for the SEM samples, the moisture content was 17.3%, the curing time was 14 d, and the xanthan gum biopolymer contents were 0%, 0.5%, 1%, 1.5%, 2%, and 3%.

## 3. Cumulative Strain of Stabilized Soil Under Traffic Loading

In this section, the effects of xanthan gum biopolymer content, curing time, moisture content, confining pressure, and cyclic stress amplitude on cumulative strain are analyzed. 

### 3.1. Influence of Xanthan Gum Biopolymer Content on Cumulative Strain

**Figure 6:**
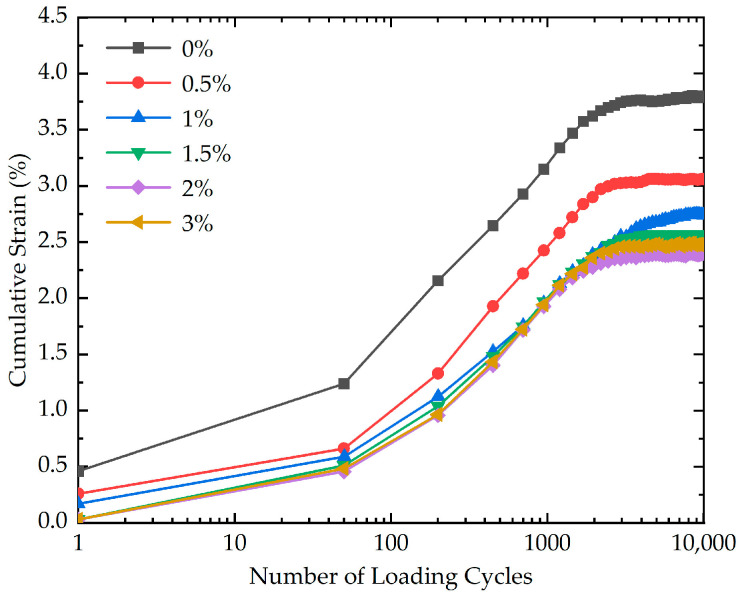
The effect of xanthan gum biopolymer content on the cumulative strain of stabilized soil.

**Figure 7:**
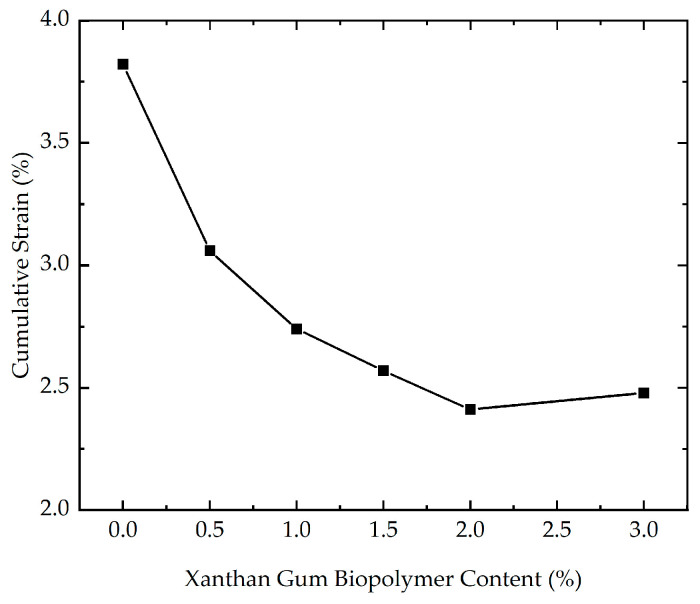
The cumulative strain of stabilized soil with different xanthan gum biopolymer contents at the end of loading.

The impact of xanthan gum biopolymer content on the cumulative strain was studied by six cyclic loading tests with different xanthan gum biopolymer contents (0%, 0.5%, 1%, 1.5%, 2%, and 3%). The effect of xanthan gum biopolymer content on the cumulative strain is demonstrated in Figure 6. As presented in Figure 6, the cumulative strain is strongly influenced by xanthan gum biopolymer content. In addition, before the number of loading cycles reaches 2000, the growth rate of the cumulative strain is higher. This phenomenon may be explained by the fact that before loading, the soil’s internal structure is relatively loose. At the initial stage of loading, specifically when the number of loading cycles is less than 2000, the soil skeleton undergoes rapid compaction, resulting in a quick increase in cumulative strain. As loading progresses, the soil particles rearrange, leading to a stable and relatively dense structure, with little change in cumulative strain. The cumulative strain of stabilized soil with different xanthan gum biopolymer contents at the end of loading is illustrated in Figure 7. As highlighted in Figure 7, the cumulative strain decreases as the xanthan gum biopolymer content increases. Notably, the cumulative strain corresponding to the xanthan gum biopolymer content of 2% is almost equal to that corresponding to the xanthan gum biopolymer content of 3%. From an economic perspective, it is recommended that the xanthan gum biopolymer content is 2% in practical engineering. One reason that can explain this phenomenon is that for the specimen with a higher xanthan gum biopolymer content, the strong inter-particle bonds are better able to resist the loading, maintaining the structural integrity of the soil. This increased resistance to structural damage helps reduce axial deformation and results in lower cumulative strain.

### 3.2. Influence of Curing Time on Cumulative Strain

**Figure 8:**
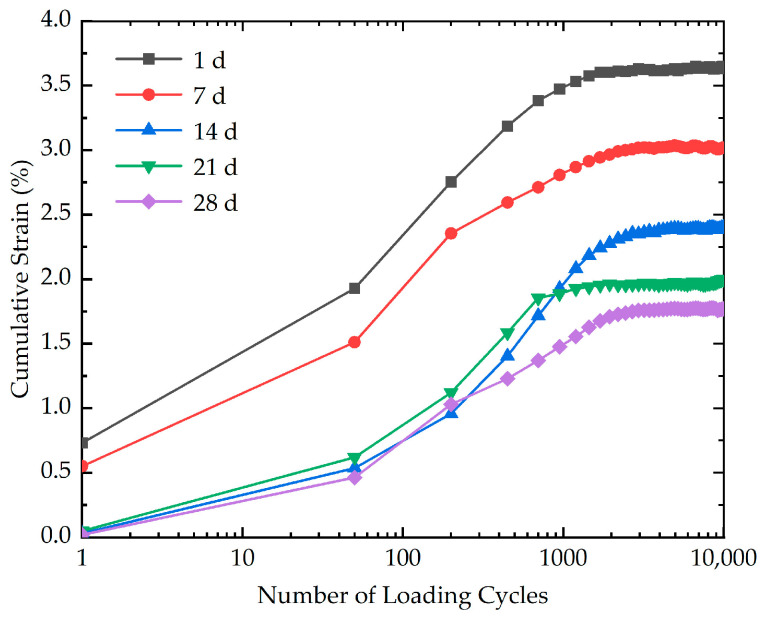
The effect of curing time on the cumulative strain of stabilized soil.

**Figure 9:**
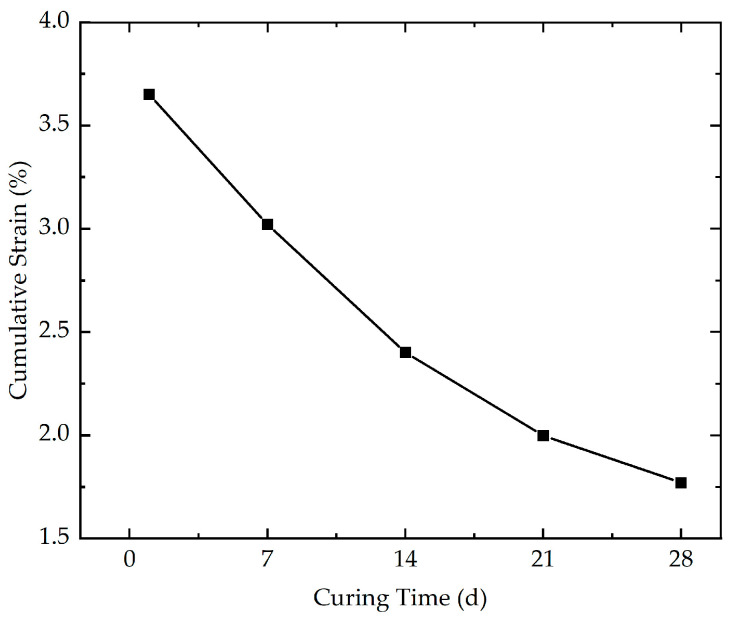
The cumulative strain of stabilized soil with different curing times at the end of loading.

With the aim of evaluating the effect of curing time on the cumulative strain, five cyclic loading tests with different curing times (1 d, 7 d, 14 d, 21 d, and 28 d) were performed. The effect of curing time on the cumulative strain is indicated in Figure 8. As depicted in Figure 8, the results demonstrate that the curing time has a significant effect on the cumulative strain. It is evident that for a specific number of loading cycles, a shorter curing time results in greater cumulative strain. The cumulative strain of stabilized soil with different curing times at the end of loading is demonstrated in Figure 9. As evidenced in Figure 9, it is obvious that the cumulative strain decreases from 3.65% to 1.77% as the curing time increases from 1 d to 28 d, indicating a 51.5% decline in cumulative strain. A possible reason for this phenomenon is that the shorter curing time may not allow for the complete reaction between the xanthan gum biopolymer and water, resulting in a less developed and weaker inter-particle bonding. The insufficient formation of cementation bridges renders the specimen more susceptible to deformation and structural damage, leading to greater cumulative strain and reduced stability.

### 3.3. Influence of Moisture Content on Cumulative Strain

**Figure 10:**
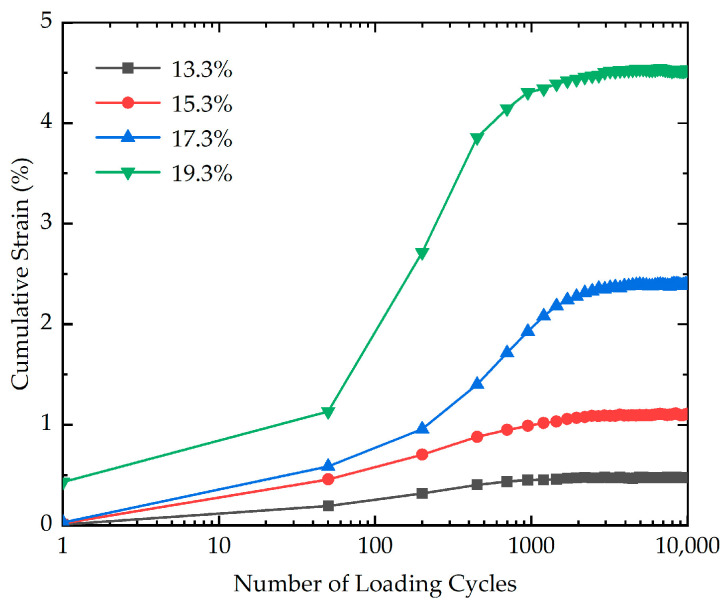
The effect of moisture content on the cumulative strain of stabilized soil.

**Figure 11:**
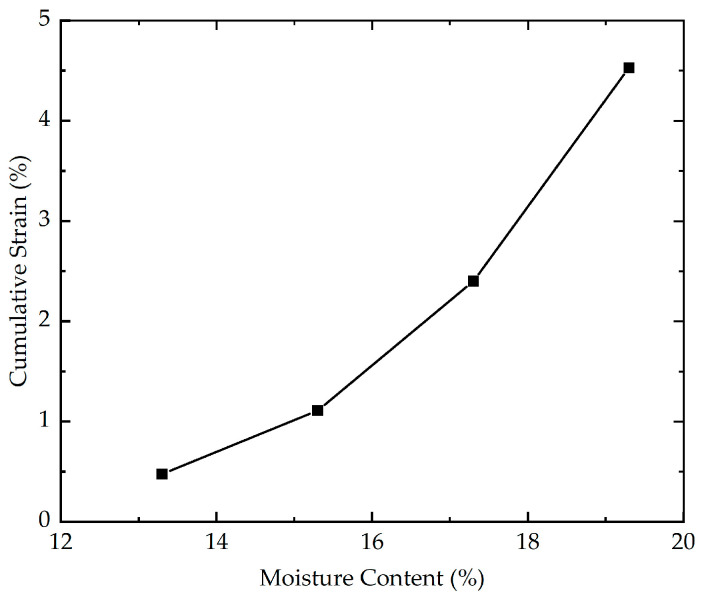
The cumulative strain of stabilized soil with different moisture contents at the end of loading.

To assess the influence of moisture content on the cumulative strain, five cyclic loading tests with different moisture contents (13.3%, 15.3%, 17.3%, and 19.3%) were carried out. The effect of moisture content on the cumulative strain is shown in Figure 10. According to the measured results demonstrated in Figure 10, for the stabilized soil with higher moisture contents (17.3% and 19.3%), there is a considerable rise in the growth amplitude of the cumulative strain with the increasing moisture content. In contrast, for the stabilized soil with lower moisture contents (13.3% and 15.3%), the cumulative strain exhibits a relatively modest increment with the increasing moisture content. The cumulative strain of stabilized soil with different moisture contents at the end of loading is presented in Figure 11. As illustrated in Figure 11, as the moisture content increases from 13.3% to 19.3%, the cumulative strain experiences a staggering 853% increase at the end of loading. The results may be due to the fact that at higher moisture content, the water molecules can form a thicker film around the soil particles, effectively reducing the contact area between adjacent particles. This reduction in inter-particle contact area weakens the soil structure and decreases the resistance to deformation. As a consequence, the reinforced soil with a higher moisture content is more easily compressed. 

### 3.4. Influence of Confining Pressure on Cumulative Strain

**Figure 12:**
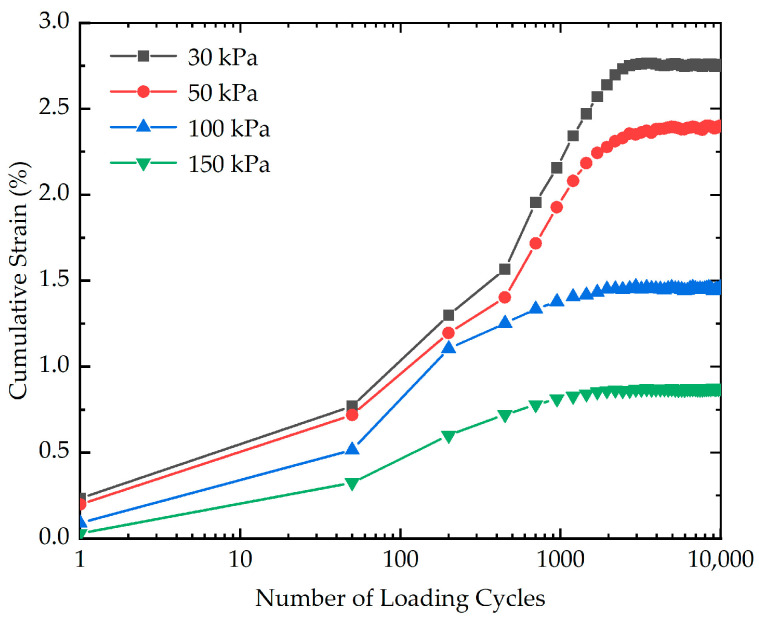
The effect of confining pressure on the cumulative strain of stabilized soil.

**Figure 13:**
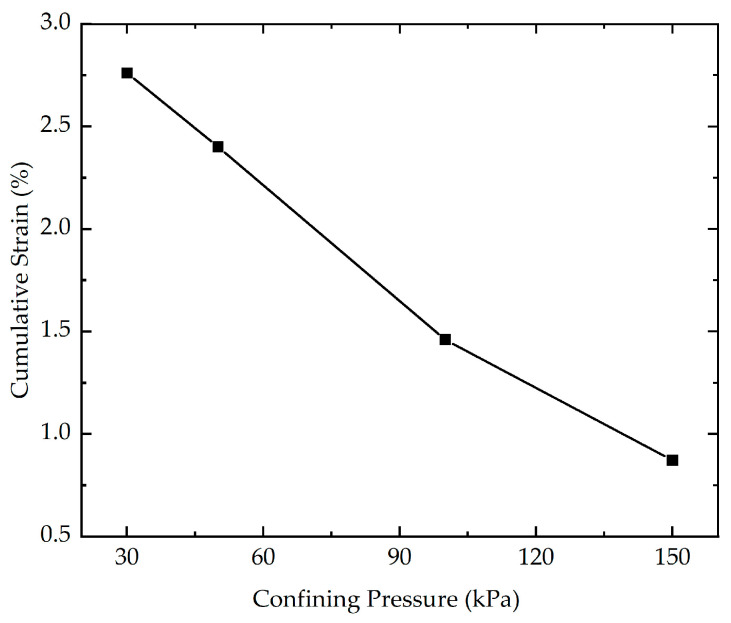
The cumulative strain of stabilized soil with different confining pressures at the end of loading.

In order to study the influence of confining pressure on the cumulative strain, four cyclic loading tests with different confining pressures (30 kPa, 50 kPa, 100 kPa, and 150 kPa) were carried out. The effect of confining pressure on the cumulative strain is presented in Figure 12. As indicated in Figure 12, the confining pressure has a substantial impact on the cumulative strain. For a given number of loading cycles, the greater the confining pressure, the smaller the cumulative strain. The cumulative strain of stabilized soil with different confining pressures at the end of loading is illustrated in Figure 13. As highlighted in Figure 13, the cumulative strain decreases from 2.76% to 0.87% as the confining pressure increases from 30 kPa to 150 kPa. The possible reason for this phenomenon is that under higher confining pressures, the soil particles experience greater inter-particle contact forces, which promote the formation of a denser and more structured soil fabric with stronger inter-particle bonding. The more compact and cohesive arrangement of particles can give rise to an increase in the capability of resisting deformation and failure, resulting in the higher the confining pressure, the lower the cumulative strain. 

### 3.5. Influence of Cyclic Stress Amplitude on Cumulative Strain

**Figure 14:**
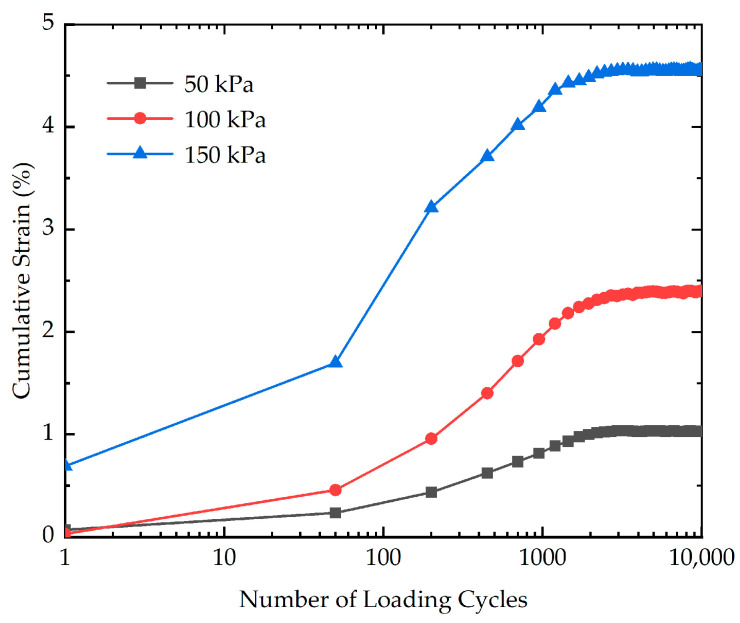
The effect of cyclic stress amplitude on the cumulative strain of stabilized soil.

**Figure 15:**
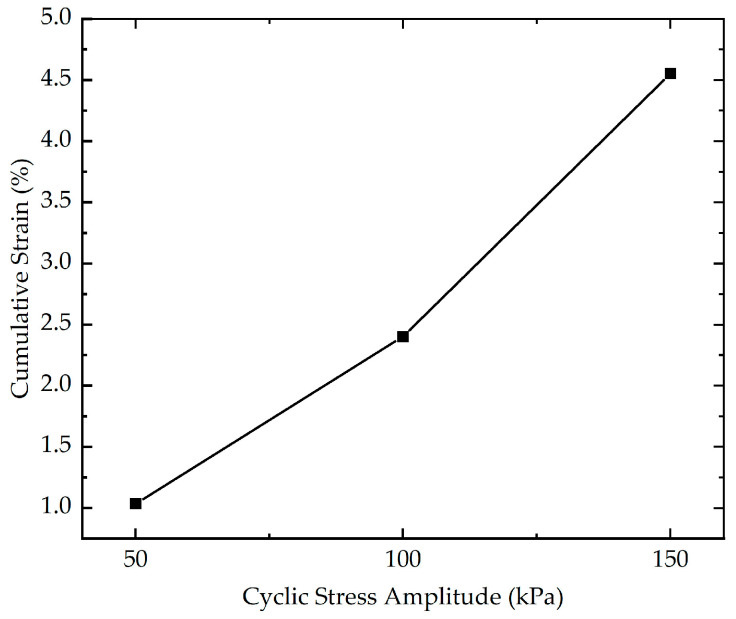
The cumulative strain of stabilized soil with different cyclic stress amplitudes at the end of loading.

To investigate the impact of cyclic stress amplitude on the cumulative strain, three cyclic loading tests with different cyclic stress amplitudes (50 kPa, 100 kPa, and 150 kPa) were performed. The effect of cyclic stress amplitude on the cumulative strain is indicated in Figure 14. As evidenced in Figure 14, the results reveal that the cyclic stress amplitude exerts a major influence on the cumulative strain. During the initial phase of loading, for the stabilized soil with higher cyclic stress amplitude, the rate of increase in cumulative strain is greater. The cumulative strain of stabilized soil with different cyclic stress amplitudes at the end of loading is presented in Figure 15. As demonstrated in Figure 15, the cumulative strain exhibits an almost linear increasing trend with the increasing cyclic stress amplitude. The cumulative strain increases from 1.03% to 4.55% as the cyclic stress amplitude increases from 50 kPa to 150 kPa. This phenomenon may be explained by the fact that when subjected to long-term, high-level cyclic loading, the soil skeleton, which forms a load-bearing framework, can undergo significant deterioration and damage. As the loading progresses, the increasing damage in the soil skeleton caused by cyclic loading can reduce the load-bearing capacity, making it more vulnerable to deformation and compression. As the inter-particle bonds break down, the soil becomes less able to maintain structural integrity, leading to a significant reduction in its resistance to cyclic loading. 

## 4. Cumulative Strain Model

### 4.1. Improved Barksdale Model

The cumulative strain of soil is frequently described by the Barksdale model, as indicated in Equation (1).


(1) ε=A+BlogN

**Figure 16:**
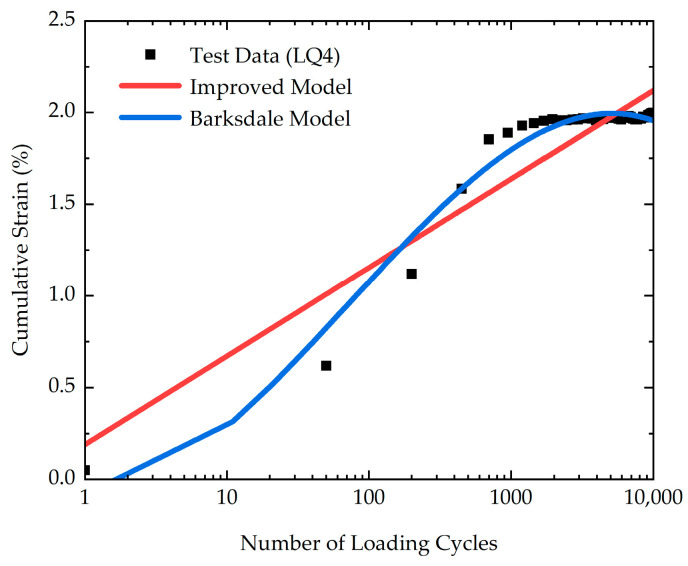
The comparison of the test data with the calculated results of the Barksdale model and the improved model.

Regrettably, the Barksdale model does not accurately represent the experimental data in this study, as indicated in Figure 16. The R^2^ of the fitting curve of the Barksdale model is only 0.819. As a result, the Barksdale model needs to be improved to accurately capture the characteristics of cumulative strain. In this research, based on the Barksdale model, the improved model is expressed as Equation (2) [30]. The comparison of the test data with the calculated results of the Barksdale model and the improved model is depicted in Figure 16. The R^2^ of the fitting curve of the improved model is 0.97.


(2) $$\varepsilon =A+B\log N+C{\left(\log N\right)}^{2}$$

### 4.2. Model Calibration

**Figure 17:**
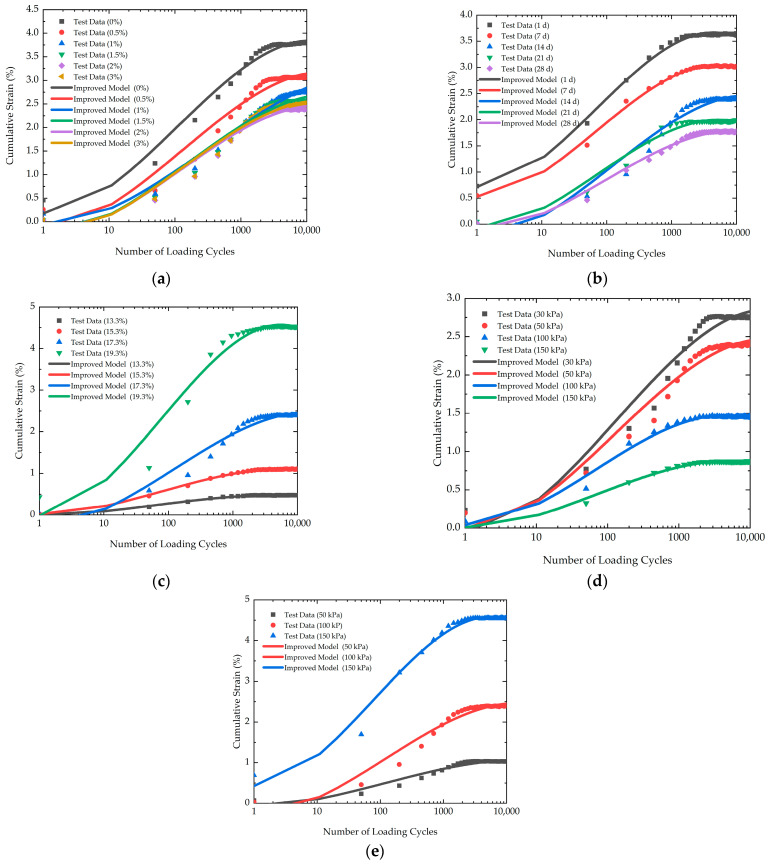
The calibration of the improved model: (**a**) CL1-CL6; (**b**) LQ1-LQ5; (**c**) HSL1-HSL5; (**d**) WY1-WY4; (**e**) FZ1-FZ3.

To calibrate the improved model proposed in this study, the experimental data and the calculated results of the improved model are compared in Figure 17. The model parameters are listed in Table 4. According to Figure 17, it is evident that the calculated results of the improved model closely agree with the laboratory data. Consequently, the improved model proposed in the current study provides a better description of cumulative strain characteristics. 

## 5. Improvement Mechanisms of Soil Reinforced by Xanthan Gum Biopolymer

**Figure 18:**
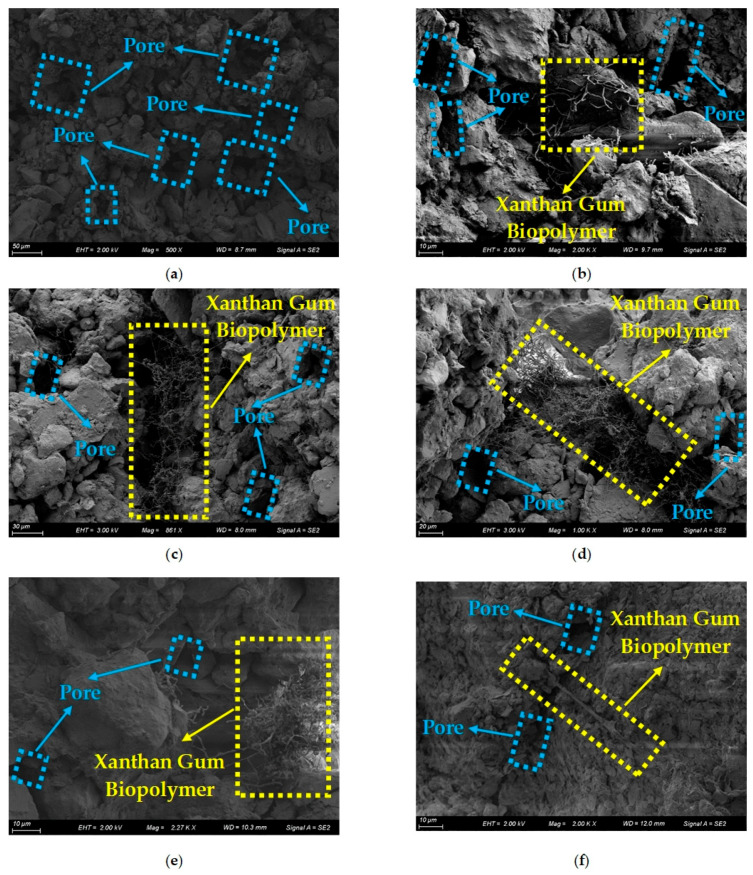
The SEM images of xanthan gum biopolymer treated soil: the xanthan gum biopolymer contents of (**a**–**f**) are 0%, 0.5%, 1%, 1.5%, 2%, and 3%, respectively.

As mentioned previously, the improvement mechanisms of soil reinforced by xanthan gum biopolymer can be obtained by analyzing the SEM images. The SEM images of xanthan gum biopolymer-treated soil with different xanthan gum biopolymer contents (0%, 0.5%, 1%, 1.5%, 2%, and 3%) are presented in Figure 18. As indicated in Figure 18a, for the soil with a xanthan gum biopolymer content of 0%, the soil displayed a relatively weak and loose microscopic structure, with distinct boundaries between individual soil aggregates. Furthermore, the soil exhibited a high quantity and size of pores, without any attached substances. 

As illustrated in Figure 18b, for the stabilized soil with a xanthan gum biopolymer content of 0.5%, a “branch-like” hydrogel can be formed when xanthan gum biopolymer interacts with water. It was then observed that the bridging structures were found between the distant soil particles that were not directly in contact, facilitated by the adsorption and pulling effect of the “branch-like” hydrogel. Additionally, xanthan gum biopolymer formed a biofilm around the soil particles, resulting in the agglomeration of soil particles. Simultaneously, the biofilm coating on soil particles expanded the contact area between the soil particles.

As demonstrated in Figure 18c,d, for the stabilized soil with xanthan gum biopolymer contents of 1% and 1.5%, xanthan gum biopolymer can generate more hydrogel when encountering water, and its shape transitions from the “branch-like” to the dense “net-like” as the xanthan gum biopolymer content increases. Subsequently, the dense “net-like” hydrogel not only enveloped the surrounding soil particles but also filled the pores, making the boundaries between the soil particles less distinct. In particular, the dense “net-like” hydrogel strengthened the bonding properties and lessened the fluidity of soil particles, leading to a beneficial increase in overall compactness. 

As depicted in Figure 18e, for the stabilized soil with a xanthan gum biopolymer content of 2%, the reaction of xanthan gum biopolymer with water leads to the “flocculent” hydrogel. It is worth mentioning that the “flocculent” hydrogel features a denser structure and greater bonding strength in comparison to the “branch-like” and “net-like” hydrogels. Because of this, the “flocculent” hydrogel can efficiently cover the surrounding soil particles, resulting in a larger contact area between them. The “flocculent” hydrogel can also firmly cement the soil particles together, enhancing soil cohesion and stability. In addition, the “flocculent” hydrogel can fill the pores in the stabilized soil, minimizing the movement of soil particles and thus reducing soil deformation. 

As evidenced in Figure 18f, for the stabilized soil with a xanthan gum biopolymer content of 3%, xanthan gum biopolymer reacting with water results in a large amount of biofilm stacked on the soil surfaces. Meanwhile, the shape of the hydrogel changes from “flocculent” to “belt-like”. This phenomenon indicates that higher xanthan gum biopolymer content is capable of generating more hydrogel, leading to a thicker adhesive bond between the soil particles. However, it is crucial to point out that compared to the “flocculent” hydrogel, the “belt-like” hydrogel, if stacked excessively, can reduce the effective contact area and increase the distance between the soil particles. Furthermore, the “belt-like” hydrogel is not effective in filling the pores in the stabilized soil. Hence, the cumulative strain with a xanthan gum biopolymer content of 3% is greater than that with a xanthan gum biopolymer content of 2%.

## 6. Conclusions

In order to accurately investigate the cumulative strain and improvement mechanisms of soil reinforced by xanthan gum biopolymer under traffic loading, a series of cyclic triaxial tests and SEM tests were performed. Based on the test data, the following conclusions can be drawn:

1. For specimens with a higher xanthan gum biopolymer content, the strong inter-particle bonds are better able to resist the loading, maintaining the structural integrity of the soil. The cumulative strain decreases from 3.65% to 1.77% as the curing time increases from 1 d to 28 d, indicating a 51.5% decline in cumulative strain.
2. As the moisture content increases from 13.3% to 19.3%, the cumulative strain experiences a staggering 853% increase at the end of loading. During the initial phase of loading, for the stabilized soil with higher cyclic stress amplitude, the rate of increase in cumulative strain is greater.
3. The improved Barksdale model is proposed to describe the experimental data in the current study. The calculated results of the improved model agree reasonably well with experimental data.
4. The “flocculent” hydrogel can firmly cement the soil particles together, enhancing soil cohesion and stability. In addition, the “flocculent” hydrogel can fill the pores in the stabilized soil, minimizing the movement of soil particles and thus reducing soil deformation.

## Figures and Tables

**Table 1:** The physical properties of soil.

Moisture Content (%)	Maximum Dry Density (g/cm^3^)	Liquid Limit (%)	Plastic Limit (%)
17.3	1.796	22.47	15.11

**Table 2:** The basic properties of xanthan gum biopolymer.

Appearance	Viscosity (1% Solution in 1% KCL)	pH (1% Solution)	Total Plate Count
white powder	1200–1800 cP	6.0–8.0	not more than 5000 cfu/g

**Table 3:** The details of the experimental scheme of cyclic triaxial test.

Sample Number	Xanthan Gum Biopolymer Content (%)	Curing Time (d)	Moisture Content (%)	Confining Pressure (kPa)	Cyclic Stress Amplitude (kPa)
CL1	0				
CL2	0.5				
CL3	1	14	17.3	50	100
CL4	1.5				
CL5	2				
CL6	3				
LQ1		1			
LQ2		7			
LQ3	2	14	17.3	50	100
LQ4		21			
LQ5		28			
HSL1			13.3		
HSL2			15.3		
HSL3	2	14	17.3	50	100
HSL4			19.3		
WY1				30	
WY2	2	14	17.3	50	100
WY3				100	
WY4				150	
FZ1					50
FZ2	2	14	17.3	50	100
FZ3					150

**Table 4:** The improved model parameters.

Sample Number	*A*	*B*	*C*	*R* ^2^
CL1	0.17303	0.66808	−0.10976	0.97692
CL2	−0.12429	0.55101	−0.08673	0.95109
CL3	−0.07184	0.38934	−0.05181	0.97433
CL4	−0.25959	0.47282	−0.07277	0.95978
CL5	−0.26891	0.47241	−0.07559	0.95258
CL6	−0.2768	0.47837	−0.07545	0.95127
LQ1	0.71129	0.66573	−0.12158	0.9934
LQ2	0.52617	0.55552	−0.10046	0.9953
LQ3	−0.26891	0.47241	−0.07559	0.95258
LQ4	−0.08316	0.45274	−0.08135	0.97021
LQ5	−0.1121	0.36633	−0.06193	0.98192
HSL1	0.0015	0.107	−0.01947	0.99156
HSL2	0.01679	0.23018	−0.04073	0.99774
HSL3	−0.26891	0.47241	−0.07559	0.95258
HSL4	−0.01894	0.98362	−0.17533	0.95592
WY1	−0.06039	0.48982	−0.07729	0.94701
WY2	−0.26891	0.47241	−0.07559	0.95258
WY3	0.0378	0.32557	−0.0597	0.98107
WY4	0.00436	0.19192	−0.03461	0.99177
FZ1	−0.06268	0.19428	−0.03104	0.94229
FZ2	−0.26891	0.47241	−0.07559	0.95258
FZ3	0.42667	0.89116	−0.15881	0.98165

## Data Availability

The original contributions presented in this study are included in the article. Further inquiries can be directed to the corresponding author.
